# Clinical and Therapeutic Notes

**Published:** 1887-11-19

**Authors:** C. R. Illingworth


### Clinical and Therapeutic Notes.

By C. R. Illingworth, M.D., M.R.C.S.

### II.—Blood Corpuscles and Fibrin.

Before passing on to the consideration of inflammation and the production of fibrin, it will be necessary to refer to another element in the composition of blood, viz., that of the white corpuscles, or *leucocytes*. These are much less numerous than the red, the proportion being about 1 to 500. They consist of that elementary tissue known as protoplasm, and they have no cell-wall like the red corpuscles. Contained in the protoplasm is a mass of granular matter, more evident in some than in others. In common with all protoplasmic material, leucocytes possess the power of altering their shape. They may be observed under the microscope, when kept at a proper temperature, to contract and dilate, and to shoot out processes of various lengths, which are again withdrawn more or less completely. Movements of this description have been long known as *amœboid*, and in the living white blood corpuscle they have an especial attraction, in that by such movements many scientists believe they are endowed with the power of passing through the walls of the finest blood-vessels, and of thus taking part in inflammatory processes. This passage through the capillary walls is, however, ridiculed by other eminent authorities. Lister, whose researches are deserving always of the greatest attention, does not deny that they do wander from the blood-vessels, but he does deny that they have anything to do with inflammation and suppuration; and for this reason, that pus corpuscles, instead of being granular and devoid of cell-wall, are not granular, have distinct nuclei, and are provided with proper cell-walls.

But let us now return to the subject of fibrin. The production of this, to the physician, important material has to be taken into account in all reparative processes after injury and disease, and alsoin all natural and diseased processes. Thus, when a wound is made, and blood-vessels are divided, blood-clots seal the divided ends, and serve a useful purpose by mechanically arresting hæmorrhage to a great extent. In other familiar processes again, clots fill the maternal sinuses, and the importance of preserving them from the softening influence of putrefying materials should be fully realised by the family physician. When hæmorrhage occurs from any cause into the tissues, the clot thus formed, being protected from irritating external agencies, becomes in most cases organised after absorption of its more fluid portions, and gradually united with the tissues in which it lies. Again, since the introduction of Listerism, or the antiseptic treatment of wounds, by the eminent surgeon and scientist who first demonstrated the important results to be obtained by the prevention of fermentation in surgical practice, it has become possible to secure the organisation of blood-clot in an open wound. A large and deeply-excavated ulcer of the leg, after being brought into a thoroughly aseptic condition, was scratched until sufficient blood was drawn to fill the cavity level with the surrounding skin. After coagulation had taken place under strictly antiseptic precautions, a dressing was applied, and complete rest to the limb afforded. Kept perfectly aseptic, the clot did not break down, but gradually became organised, new capillaries passing from the deeper portions through its substance and to the surface, until in due time complete organisation was evidenced by the blood-clot itself bleeding on being scratched with a needle. Triumphs such as this speak volumes for antiseptic surgery. It is, however, in diseased processes generally that the physician is especially called upon to consider the production of fibrin, and to witness the ravages which, when unchecked, it is capable of working in the delicate structures of the body.

In all inflammatory conditions, as has been already indicated, the fibrin elements are increased in the blood: not only so, but the fibrin is actually formed or produced, and it is the formation and deposition of this substance in the tissues of the inflamed parts which give rise to many of the symptoms presented. The signs of inflammation may be described as being four in number, viz., heat, redness, swelling, and pain. This definition, however, in its entirety, refers to inflammation of a more or less superficial character—a boil, for instance. But it is certain that one or more of these signs are invariably present in inflammatory conditions of any part of the body. Of these four symptoms, the physician is most concerned with the first three, because they are *objective*, the *subjective* one of pain being chiefly important from the patient's point of view. The most reliable guide to the extent and severity of an inflammatory condition is heat. A rise of temperature would hardly be expected to occur in any marked degree from an ordinary boil, although the local heat would be readily distinguishable by the touch; but when the inflammatory process attacks a lung, the thermometer readily registers a considerable increase in the heat of the blood. Taking pneumonia, therefore, as an example of an inflammatory process, this increase of temperature is most probably induced as follows: From exposure to cold or wet, or both, the surface of the body is chilled to such an extent as to lead to contraction of the peripheral circulation generally. This is generally evidenced by a *rigor*, or shivering fit. The contraction of the peripheral circulation causes concentration of the blood current upon the central organs (and particularly of that part of the body most exposed to cold). The capillary circulation of the lung thus becomes gorged, and a *stasis* of the blood in the parenchyma of the lung ensues. This excites the filaments of the vagus and sympathetic nerves, and leads to increase of the processes of respiration and circulation. But the increase of pressure put upon the delicate vascular structure of the lung has induced a condition of prostration of its vital energy, leading to the effusion of plastic lymph into the air-cells—plastic because of the globulin communicated by the stationary or slowly-moving corpuscles to the plasma. The increase of the respiratory and circulatory process by the *stasis* leads to dilatation of the systemic capillaries and increased chemical change in the tissues, all of which processes cause a rise of temperature from the normal of 98·4 deg. Fahr. to somewhere about 103 deg.

The condition of *stasis* with commencing plastic effusion into the air-cells is known as the first stage of pneumonia, or the stage of congestion. The percussion note over the affected area is higher pitched than the normal, because less air can enter the organ; the air on entering the lung disturbs the effusing lymph, and causes fine crepitations, and the vocal resonance is much increased on account of the greater conducting power of the inflamed area. When effusion to a complete filling of the air-cells with plastic material, and coagulation of the effusion occurs, we have the second stage of the disease, or *hepatisation*, so termed from the resemblance the organ now bears to the liver in its solidity. In this stage the dulness on percussion is absolute, and the respiration is of course unaccompanied by the fine crepitations of the first stage. It is easy now to perceive another element in the rise of temperature in the increased muscular action required to make the remaining portions of the lung do extra work.

It will thus appear evident that fibrin, formed and deposited in the tissues of an organ as the result of inflammatory action, is a source of great danger to the economy. The subject, therefore, of fibrin formation, and the action of certain drugs which prevent it, deserves the closest attention in the treatment of diseases which are associated with an over-production of this material. Such diseases are pneumonia, bronchitis, pleurisy, nephritis, meningitis, acute rheumatism, gastritis, etc., in all of which there is an increase of the fibrinous elements of the blood, and a consequent indication for the administration of what may be termed "liquefacient" remedies, such as ammonia, soda, the salicylates, and the iodides of potassium and sodium.

(*To be continued.*)

